# Childhood immunisation default rate and its associated factors among children attending public health clinics in Klang Health District, Selangor, Malaysia: A cross-sectional study

**DOI:** 10.51866/oa.1065

**Published:** 2026-10-13

**Authors:** Ahmad Fairol Mahat, Selvam Renuka, Palaniandi Sinthamani, Siti Nurhanani Zaik, Sathiawathy Selvarajan, Suet Yin Chow, Fadzilah Mohamad

**Affiliations:** 1 Klinik Kesihatan Kapar, Jalan Perbandaran Taman Kapar, Kapar,Klang Selangor, Malaysia.; 2 Klinik Kesihatan Pandamaran, Jalan Raja Muda Musa, Kawasan 10, Pelabuhan Klang, Selangor, Malaysia.; 3 Klinik Kesihatan Bukit Kuda, Kawasan 15, Jalan Batu Tiga Lama, Klang, Selangor, Malaysia.; 4 Klinik Kesihatan Meru, Jalan Kenangan, Klang, Selangor, Malaysia.; 5 Klinik Kesihatan Anika, Jalan Tengku Kelana, Klang, Selangor, Malaysia.; 6 IMU Medical Clinic, No 126, Jalan Jalil Perkasa 19, Bukit Jalil, Kuala Lumpur, Malaysia.; 7 Department of Family Medicine, Faculty of Medicine and Health Sciences, Universiti Putra Malaysia, UPM Serdang, Selangor, Malaysia.

**Keywords:** Immunisation programs, Default rate, Children, Vaccination, Risk factors

## Abstract

**Introduction:**

Childhood immunisation is one of the World Health Organization’s strategies for reducing vaccine-preventable diseases (VPDs). However, a proportion of children in Malaysia remain unvaccinated, as reflected in the increasing incidence of VPDs. This study aimed to determine the childhood immunisation default rate (CIDR) and its associated factors among children in Klang District.

**Methods:**

A cross-sectional study was conducted from July to September 2023 among parents or guardians accompanying children aged 12-24 months at selected maternal and child health clinics in Klang Health District. Systematic random sampling was used, and an assisted validated questionnaire was utilised to assess sociodemographic, immunisation and associated factors. Data were analysed using SPSS version 29, involving three-level analysis (descriptive, bivariate (chi- square and Fisher’s exact test) and multivariable logistic regression).

**Results:**

A total of 400 respondents were recruited. The CIDR was 25.5%. The second measles, mumps and rubella (MMR) vaccine dose (22.1%) had the highest default rate, followed by the third pneumococcal vaccine dose (16.3%) and first MMR vaccine dose (14.3%). Child with younger age group (P=0.045) was found to be a risk factor, whereby healthy children (P<0.001) and having no transportation (P<0.001) were the protective factors to defaulting.

**Conclusion:**

This study shows a high CIDR, reflecting gaps in existing policies and systems. Child’s age, health status and transportation were significantly associated with the CIDR. Therefore, more awareness and knowledge about the contraindications for immunisation and the importance of correct timing for immunisation should be emphasis. The associations between immunisations defaulting and transportation availability warrants further investigations.

## Introduction

Childhood immunisation is the safest and most effective means of protecting children from serious diseases. Since the introduction of the Expanded Programme on Immunisation (EPI) in 1974, the World Health Organization (WHO) has significantly reduced the incidence of various diseases.^1^

Despite the cost-effectiveness of vaccines,^2^ around 14.5 million children globally still miss routine immunisations.^1^ Malaysia has also adopted the EPI and achieved high immunisation coverage exceeding 90%, which aligns with the Ministry of Health target of more than 95% coverage.^3^ Despite its success, childhood immunisation uptake still faces new challenges with the increasing trend of vaccine hesitancy, leading to defaulting vaccination.^4,5^ Vaccination coverage has been inconsistent and is starting to decline. For example, the coverage for the second measles vaccine dose dropped from about 99% in 2014–2017 to around 90% in 2018.^2^ Studies have shown that the factors influencing childhood immunisation uptake include client-related, provider-related and environmental factors.^6,7^ Client-related factors include knowledge of vaccines, socioeconomic status, family size, birth order, marital status, family mobility and job stability.^8^ Provider-related factors include waiting time, motivation and attitude of service providers, timing of immunisation sessions, side effects and charges for services. Conversely, external or environmental factors include logistic barriers, access to healthcare, preparedness of the community for immunisation sessions, dissemination of information and inter-sectoral collaboration.

In Sabah, Azhar et al.^8^ found that being a working mother, living in a rural area and having a larger family size were significantly associated with defaulting. Another study by Krishna et al.^9^ conducted among children in daycare centres in Petaling District highlighted the need for intervention programmes to improve the childhood immunisation status, focusing on the nonMuslim community with a larger family size and low educational level. However, these studies covered different populations and were conducted before the COVID-19 pandemic. Post- COVID-19 service disruptions and the increasing vaccine hesitancy have made it more difficult to maintain good immunisation coverage in Malaysia.

As no studies have been conducted after the pandemic, it is important to determine the current childhood immunisation default rate (CIDR) and explore its potential associated factors to achieve better preventive measures. Hence, this study aimed to determine the CIDR and its associated factors among children attending public health clinics. Identifying these factors can help healthcare providers use a better approach in improving vaccine coverage.

## Methods

### Study design and setting

A cross-sectional study was conducted from July to September 2023 among parents or guardians accompanying children for maternal and child health clinic (MCHC) follow-up at four health clinics in Klang Health District: Klinik Kesihatan Kapar, Klinik Kesihatan Bukit Kuda, Klinik Kesihatan Pandamaran and Klinik Kesihatan Meru. Klang Health District was selected due to its high population density, diverse socioeconomic profile and established public health clinics, making it an ideal setting to study the CIDR and its associated factors.

### Inclusion and exclusion criteria

Parents or guardians of children aged 12–24 months with an immunisation record book from birth to the current age, who were able to read and understand English or Malay, were invited to join this study. However, parents or guardians of children whose immunisation record book was unavailable, was lost, was damaged or contained unclear information or those of children who were contraindicated for vaccination (e.g. immunocompromised) were excluded from this study. The age group of 12–24 months was selected because most recommended immunisations were for children aged under 12 months.

### Sample size calculation

The sample size for this study was determined using the formula for comparing two independent proportions, as originally proposed by Lemeshow et al. for the WHO. The calculation was based on the anticipated prevalence of defaulting, with P1=39.1% among those with a travel time of less than 30 minutes and P2=29% among those with a travel time of 30 minutes or more, as previously reported.^9^ The assumption included a 5% margin of error and a 95% confidence level. Given a 20% non-response rate, the sample size was inflated to 400. This sample size was then divided equally among the four clinics, resulting in 100 participants per clinic.

### Sampling method

Systematic random sampling was used. The sampling frame was the attendance list of children who attended MCHCs on the day of data collection and fulfilled the eligibility criteria. The following formula was used:


$$\text{k}=\frac{\text{N}}{\text{n}}$$

where k is the systematic sampling interval, N is the population size and n is the sample size. The average population size of children aged 12–24 months attending all four health clinics within 3 months is roughly 10,000, with an average of 2500 and a sample size of 100 in each clinic. Hence, put into the formula, every 25th patient in the list was invited to participate in this study. The site investigators identified and approached eligible participants. Written informed consent was obtained prior to enrolling them. The investigators interviewed participants using the study instrument. Information on children’s immunisation records was cross-checked with clinic records.

### Study instrument

An assisted validated questionnaire, divided into the following three parts, was used: Part A (sociodemographic details of parents or guardians of children), Part B (children’s immunisation history) and Part C (child-related factors, parent- or guardian-related factors, family-related factors and health service-related factors). This questionnaire was adopted from Azhar et al.^8^ A pretest was conducted among 30 respondents prior to the actual study to assess the feasibility of the study instrument in the local setting. The results of the pretest were excluded from the final analysis. The pretest showed that the study instrument was clear, feasible and manageable.

### Study variables

The dependent variable was the CIDR. Defaulters of childhood immunisation included children who had defaulted one or more doses of vaccination from birth to their respective age, while nondefaulters included those whose immunisation status was up to date for their age.^2^ The independent variables were the sociodemographic characteristics of respondents (age, citizenship, ethnicity, religion, marital status, highest educational level, employment status and health status), child- related factors (age, sex, birth order, place of birth and health status), family-related factors (family size, type, income and mobility) and health service-related factors (transportation, cost, waiting time and negative experience).

### Statistical analysis

Data entry and statistical analysis were performed using IBM SPSS Statistics version 29 (SPSS Inc., Chicago, IL). Descriptive analysis was used to summarise variables. Categorical variables were presented as frequencies and percentages. Normality tests were conducted for continuous variables (parental or guardian age and child age) using the Shapiro—Wilk test. Continuous data with a normal distribution were presented as means and standard deviations, whereas non-normally distributed data were described as medians and interquartile ranges (IQRs). For association analysis, age variables were categorised. The chi-square test or Fisher’s exact test, where appropriate, was used to determine the association between the CIDR and child-related, parent- or guardian- related, family-related and health service-related factors. For comparisons involving non-normally distributed continuous variables, the Mann—Whitney U test was applied. Variables with a p-value of less than 0.25 in the bivariate analysis were entered into the multivariate analysis using binary logistic regression to identify the independent predictors of the CIDR. Statistical significance was set at P<0.05.

## Results

A total of 400 parents or guardians from the four selected health clinics responded to the questionnaire, yielding a response rate of 100%.

### Respondent characteristics

The median age of the parents or guardians was 31.0 years (IQR: 8). Approximately 3.2% were non-Malaysian citizens. The majority were Malays (61%), received at least secondary education (39.5%), were married (99.5%) and were employed (59.5%).

The age of the children ranged from 12 to 24 months, with a median age of 18 months (IQR: 3).

Approximately 52% were boys. Most of them were delivered in the hospital (96.2%), and only 15 were delivered at home (3.8%). The sociodemographic characteristics are summarised in Table 1.

**Table 1:** Sociodemographic characteristics of the respondents (N=400).

Variable	Frequency	Percentage	Median (IQR)
Parent’s or guardian’s age (year)			31.0 (8.0)
Citizenship			
Malaysian	387	96.8	
Non-Malaysian	13	3.2	
Ethnicity			
Malay	244	61.0	
Non-Malay	156	39.0	
Parent’s or guardian’s educational level			
Primary school	20	5.0	
Secondary school	158	39.5	
Pre-university (STPM/A-level)	27	6.7	
Diploma	97	24.3	
Degree	88	22.0	
Postgraduate studies	10	2.5	
Parent’s marital status			
Married	398	99.5	
Unmarried	2	0.5	
Parent’s employment status			
Employed	238	59.5	
Unemployed	162	40.5	
Child’s age (month)			18.0 (3.0)
Child’s sex			
Male	210	52.5	
Female	190	47.5	
Child’s place of birth			
Hospital	385	96.2	
Home	15	3.8	

### Prevalence of defaulting and type of immunisation defaulted

Table 2 presents the prevalence of childhood immunisation uptake in the four selected public health clinics. Approximately 25.5% of the respondents were defaulters.

**Table 2:** Prevalence of childhood immunisation defaulters versus non-defaulters (N=400).

Childhood immunisation	Frequency	Percentage
Defaulter	102	25.5
Non-defaulter	298	74.5

Table 3 demonstrates the percentages of the various types of vaccinations that were defaulted by the children, calculated from the proportion of the defaulters. The highest percentage of the defaulted vaccinations was observed for the second dose of the measles, mumps and rubella (MMR) vaccine at 22.1%. This was followed by the third dose of the pneumococcal vaccine at 16.3% and the first dose of the MMR vaccine at 14.3%.

**Table 3:** Percentage of the various types of immunisation defaulted (n=102).

Type of immunisation	Recommended age given (month)	Percentage of defaulter
BCG/hepatitis B-first dose	0	3.2
Hexaxim-first dose	2	7.1
Hexaxim-second dose	3	6.5
Pneumococcal-first dose	4	4.6
Hexaxim-third dose	5	5.8
Pneumococcal-second dose	6	11.1
MMR-first dose	9	14.3
MMR-second dose	12	22.1
Pneumococcal-third dose	15	16.3
Hexaxim-booster dose	18	9.0

### Factors associated with the CIDR

Table 4 shows the results of the inferential analysis of the factors associated with the CIDR. For the child-related factors, the child’s health status was significantly associated with the CIDR (P<0.001). For the health service-related factors, transportation issues showed a significant association with the CIDR (P=0.003). There were no parent-, guardian- or family-related factors found to be associated with the CIDR.

**Table 4:** Factors associated with the childhood immunisation default rate (N=400).

Factor	Median (IQR)	Defaulter (n=102)	Non-defaulter (n=298)	P-value
**A. Child**				
**Age, month** 12-18 19-24		82 (27.0) 20 (20.8)	222 (73.0) 76 (79.2)	**0.229** ^+^
**Sex** Male Female		49 (23.3) 53 (27.9)	161 (76.7) 137 (72.1)	0.296^+^
**Birth order**	2.0 (2.0)			0.713^#^
**Place of birth** Health facility Home		99 (25.7) 3 (20.0)	286 (74.3) 12 (80.0)	0.769^^^
**Health status** Healthy Unhealthy		56 (16.7) 46 (71.9)	280 (83.3) 18 (28.1)	**<0.001** ^+^
**B. Parent or guardian**				
**Age, year** 20–30 31–40 41–50 51–60		39 (22.8) 53 (26.4) 9 (34.6) 1 (50.0)	132 (77.2) 148 (73.6) 17 (65.4) 1 (50.0)	0.475^+^
**Educational level** No formal education Primary education Secondary/tertiary education		1 (14.3) 37 (28.7) 64 (24.2)	6 (85.7) 92 (71.3) 200 (75.8)	0.504^+^
Working status Unemployed Employed		41 (25.3) 61 (25.6)	121 (74.7) 177 (74.4)	0.942^+^
Health status Healthy Unhealthy		101 (25.4) 1 (33.3)	296 (74.6) 2 (66.7)	1.000^^^
**Marital status** Married Single		101 (25.5) 1 (25.0)	295 (74.5) 3 (75.0)	1.000^^^
**C. Family**				
**Family size (number of sibling)** <5 ≥5		56 (25.5) 46 (25.6)	164 (74.5) 134 (74.4)	0.982^+^
**Family type** Nuclear Extended		164 (74.5) 134 (74.4)	233 (73.0) 65 (80.2)	**0.184** ^+^
**Family income** <RM 4850 RM 4850–10,959 >RM 10,960		40 (24.5) 31 (22.8) 31 (30.7)	123 (75.5) 105 (77.2) 70 (69.3)	0.361^+^
**Family mobility** Yes No		16 (32.0) 86 (24.6)	34 (68.0) 264 (75.4)	0.260^+^
**D. Health service**				
**Transportation** Yes No		24 (41.4) 78 (22.8)	34 (58.6) 264 (77.2)	**0.003** ^+^
**Cost** Yes No		12 (30.0) 90 (25.0)	28 (70.0) 270 (75.0)	0.491^+^
**Waiting time** Yes No		22 (25.9) 80 (25.4)	63 (74.1) 235 (74.6)	0.927^+^

+ Pearson chi-square test

^ Fisher’s exact test

# Mann–Whitney U test; *Significant at P<0.05.

Table 5 shows the results of the multivariable logistic regression for the predictors of defaulting. The regression analysis showed that three factors were significant independent predictors: Child’s age, healthy child status and unavailability of transportation to healthcare facilities. From this study, it was found that child’s age was a risk to defaulting where younger child (12-18 month old) was seen to have 97.1% increase in the odds of defaulting (AOR=1.97, 95% CI [1.02, 3.82], P=0.045) compared to older child with age group of 19-24 month old. However the child’s health status and transportation were found to be protective factors. Having a healthy child was associated with a 92.9% reduction in the odds of defaulting (A0R=0.07, 95% CI [0.04, 0.13], P<0.001) compared to having an unhealthy child. Whereby, patients with no transportation was found to be associated with a 67.5% reduction in the odds of defaulting (A0R=0.33, 95% CI [0.17,0.62], P<0.001) compared to those with transportation. Family type was not a significant predictor of defaulting (P=0.239). Although the odds ratio for nuclear families was 1.50, analysis found that there was no significant difference in defaulting likelihood between the nuclear and extended family types (P=0.239).

**Table 5:** Multivariable logistic regression analysis of the predictors of defaulting.

Variable	B	Std. error	AOR (95% CI)	P-value
**Child’s age** 12-18 months 19-24 months	0.68	0.338	1.97 [1.02, 3.82] 1 (Ref)	**0.045** ^*^
**Child’s health status** Healthy Unhealthy	-2.64	0.32	0.07 [0.04, 0.13] 1.00 (Ref)	**<0.001** ^*^
**Family type** Nuclear Extended	0.40	0.35	1.50 [0.76, 2.94] 1.00 (Ref)	0.239
**Health service (transportation)** No Yes	-1.12	0.33	0.33 [0.17, 0.62] 1.00 (Ref)	<0.001^*^

Reference category: Non-defaulter

B: regression co-efficient; AOR: Adjusted odds ratio; CI: Confidence interval;

* Significant at P<0.05.

## Discussion

This study found a default rate of 25.5%, which is higher than that reported in other studies (16.8%^8^ and 20.7%^9^). However, it is relatively lower than that observed in studies conducted in some African countries (28.3%^10^ and 62.8%^11^), where logistics, culture and religion may have played a substantial role. The findings of this study provide critical insights into the factors influencing clinic attendance for childhood vaccination among children attending public health clinics in Klang Health District. Our multivariate analysis identified child’s age, child health status and transportation as predictors of immunisation schedule adherence.

Our study found that child’s age was significantly associated with defaulting immunisation. Children aged 12 to 18 months had 97.1% higher odds of defaulting compared with older children aged 19 to 24 months. These findings showed similar pattern with Krisha et al^9^ in which the study location was similar with our study: Selangor urban areas - Klang district and Petaling district respectively. Both our study and Krisha et al found that parents or caregivers of younger children were more likely to default on routine immunisations.^9^ In general, younger children require more frequent immunisation visits, unpredictable schedules of sleep routines and more frequent feeding compared to older children. These could be the reasons to increasing missed appointments among younger children. Previous research has also shown that the high frequency and complexity of vaccinations visit during the first two years of life, making it difficult for parents to keep up. This led to missed visits as the schedule grows.^22^ On the other hand, the study by Azhar et al. in Sabah did not identify child’s age as an independent predictor of immunisation defaulting, as defaulting was mostly linked to maternal factors like working status and family size.^8^ However, their study still noticed that the highest proportion of defaulting were the MMR vaccines (12 months) and DPT-OPV booster (18 months). This highlighted that the period between 12 to 18 months should be given extra attention as this is the period where defaulting is high. Targeted appointment reminders and active follow-up by healthcare providers may benefit this age group to ensure timely attendance for scheduled immunisation visits.

Apart from the finding above, health status of the child was found to be significantly associated with defaulting immunisation. From the result, it was demonstrated that children reported to be healthy had 93% reduction of defaulting compared with those reported to be unhealthy. In this study, a healthy child was referred to as a child without any reported illness at the time of assessment, whereas an unhealthy child referred to a child with any reported acute or chronic illness as informed by the parent or guardian during data collection. Our study finding was consistent with the findings with previous studies.^12,13,19^ Children with reported unwell at the time of scheduled immunisation were more frequently observed among defaulters, suggesting an association between perceived poor child health and delayed or missed vaccination, likely influenced by parental misconceptions that even minor illnesses are contraindications to immunisation, despite evidence that mild illness does not preclude vaccination. Ahmad et al. found that incomplete primary immunisation was associated with frequent illness episodes and parental concerns about children’s condition, which may lead parents to postpone or skip the clinic visits.^12^ Similarly, the Ethiopian case control study by reported that children who defaulted from completing their immunisation series were more likely to have had prior illness episodes, poor health status or complications perceived by parents as reasons to delay vaccination.^13^ A Malaysian mixed method study conducted at a child development centre in Klang Valley found that children with special needs (chronic illnesses, developmental delays or multiple comorbidities) were more likely to miss scheduled appointments.^19^ This reflects a key barrier: unpredictable health crises in children with special needs disrupt attendance, triggering extended delays for rescheduling amid high demand and limited slots.

Interestingly, our study suggested that the children whose caregivers reported no access to transportation had lower odds of defaulting immunisation compared to those with access to transportation. This finding is unusual because the expected relationship would generally be having no transportation causes difficulty in reaching the clinic, therefore defaulting immunisation. Instead, our study showed otherwise. There are several plausible explanations. Firstly, transportation access may be acting as a proxy for other factors like work, other responsibilities/chores, commitment to other children, living further from the health facility, or attending different healthcare facilities. Secondly, caregivers might have transportation while still experiencing substantial barriers to attending the clinic as it does not measure distance to clinic, travelling time, traffic, transport cost, availability of the vehicle at the time of appointment, whether the caregiver can take time off work, convenience of clinic hours. Thirdly, it could be due to sampling issues as well. Of those 58 children with transportation access, 24 were defaulters, giving the 41.4% proportion. However, due to the small number of groups, the observed association may be influenced by characteristics concentrated within that group. Our study findings clearly suggest that transportation availability does not equivalent to healthcare accessibility. Having a vehicle does not necessarily mean easy access to immunisation. Traffic, parking, travelling time, distance and clinic accessibility may remain barriers. In Klang Valley, transportation barriers were reported even among caregivers using private vehicles.^19^ Other practical barriers may be more important such as work commitments, long waiting time, forgetting appointments, child illness, and appointment scheduling. These factors have been reported among Malaysian families.^17,19^ Besides that, transportation may be confounded by socioeconomic or family factors. In Sabah, transportation was associated with defaulting in bivariable analysis but was not an independent predictor after adjustment.^8^ Finally, travelling time may be more relevant than transport availability. In Petaling District, Selangor, travelling more than 30 minutes to the immunisation facility was independently associated with higher odds of defaulting.^9^

In this study, parental or guardian characteristics such as marital status, age, employment status, health status and educational level were not significantly associated with immunisation defaulting in this study. These results contrast with those reported by Krishna et al.,^9^ who found significant associations between immunisation defaulting and maternal age, religion and ethnicity, suggesting that the determinants of defaulting may vary across different settings and populations. Our study findings suggest that the immunisation default rate differs across all types of parental or guardian background including age, religion, ethnicity and educational level. A Cochrane qualitative evidence synthesis revealed that parents’ views and practices regarding childhood vaccinations were complex and dynamic regardless of background. It concluded that multiple webs of social processing and social interaction influence parents’ decision on childhood immunisation.^14^ In our model, family type was not a significant predictor. This suggests that whether a child comes from a nuclear or extended family may not be as critical as the child’s health status or clinic accessibility. While the international literature indicates that larger families or single-parent households may face logistical challenges, our findings show that child health overrides family structure in this clinical population. These results align with those of Abenova et al.,^21^ who found that household income and distance were not significant predictors of missed appointments at a Malaysian child development centre, indicating equitable access across family backgrounds. This finding contrasts with the reports by Azhar et al.,^8^ Pearce et al.^15^ and Oliveira et al.,^16^ who linked larger family sizes with higher default rates, suggesting variability in familial influences on immunisation behaviours. Cost was also not a major factor in this study, likely due to the availability of nearby immunisation facilities, free public transport and the provision of free vaccinations by government clinics. In Malaysia, immunisation services are provided by both government and private healthcare facilities. The primary healthcare system in Malaysia is recognised as exemplary for other developing countries. The World Health Report 2000 ranked Malaysia as number 49 among 191 WHO member countries in the overall health system performance.^20^ This finding contrasts with that of Azhar et al.,^8^ who identified cost as a significant factor, and with the reports by Lim et al.^17^ and Abdulraheem et al.,^11^ who found waiting time and cost to be significant factors. Nonetheless, the differences in these findings may be attributed to variations in study settings. Azhar et al.^8^ conducted their research in Sabah, a northern part of Borneo; Krishna et al.^9^ covered childcare centres; and Lim et al.^17^ and Abdulraheem et al.^11^ conducted their studies in government healthcare centres, with Lim et al.^17^ covering a larger sample size across Lembah Kinta and Abdulraheem et al.^11^ focusing on clinics in Nigeria.

The uptake of the MMR vaccine remains controversial, as it ranked the highest type of childhood vaccine to be defaulted. This indicates that some parents or guardians may still be sceptical towards MMR vaccines regardless of the first or second dose. This vaccine hesitancy is probably influenced by the misperception of vaccines as dangerous,^4^ parents’ concern over vaccine safety^11^ and fear of developing autism.^18^ Hence, correcting the misconception of childhood vaccinations, especially MMR vaccines, should be the focus topic during public programmes on vaccine information both within and beyond healthcare centres.

### Strengths and limitations

This study is the first to be conducted in Klang Health District and in a primary care setting that covers different groups within the community. It shows an overview of the childhood vaccination status, reasons for defaulting and association between defaulting immunisation and various factors. The findings also offer valuable insights for informing public health policies, developing strategies to improve vaccination coverage and reduce gaps and establishing baseline data for future longitudinal research.

However, the data may have experienced snapshot bias, potentially overlooking fluctuations or seasonal variations in immunisation practices. Although our study attempted to minimise recall bias on the immunisation records by checking the clinic records, there is still potential for this bias, as inaccuracies in remembering the reasons for defaulting might have impacted the findings. Selection bias may also be present, as the study was conducted among children attending MCHCs only, potentially leading to underestimation or overestimation of the true default rate. The CIDR may be underestimated or overestimated due to clinic-based sampling. These biases, including selection bias, were reduced by conducting the multiple logistic regression analysis. Additionally, the study findings might not be generalisable to other settings or regions, as the samples were only from four primary health clinics and may not represent the larger population.

### Recommendations

An effective vaccine reminder system, such as SMS or WhatsApp messages, can be implemented with particular attention to children aged 12-18 months to encourage the appropriate timing of vaccine administration. Education for parents and healthcare workers on the absolute and relative contraindications for immunisation might also help reduce the defaulter rate. Future studies should include local qualitative studies to explore. The possible reasons for defaulting on immunisation should be clearly documented in patient notes to reduce recall bias. Local qualitative studies should be considered to explore parents’ or guardians’ perceptions, acceptance, barriers and values placed on childhood immunisation. Future research should aim for larger and more diverse sample sizes. and explore the possible reasons for defaulting specific vaccinations.

## Conclusion

This study found a high CIDR, which suggests potential gaps in existing policies and service delivery systems. Younger age group, and healthy child status were significantly associated with the CIDR. Children aged 12-18 months had higher odds of defaulting compared with children aged 19-24 months. Therefore, healthcare providers should promote programmes aiming to increase awareness and knowledge about the absolute and relative contraindications for immunisation, with particular attention to younger children. The unexpected association between transportation availability and immunisation defaulting warrants further investigation.

## Data sharing statement

The datasets collected and analysed during the study are available upon reasonable request from the corresponding author and are subject to data protection laws and regulations.

**How does this paper make a difference in general practice?**

- This study enhances awareness among primary care doctors to re-evaluate how they can communicate the importance of immunisations, manage follow-up with patients and improve how they educate parents.
- By showing that a child’s health as a key reason for missing vaccinations, doctors can focus on helping parents with these issues. They can find ways to support parents, especially if they are worried about their child’s health and unsure about whether to get them vaccinated.
